# Performance of the IR Biotyper, Nanopore, and Illumina sequencing to discriminate *Escherichia coli* strains originating from poultry

**DOI:** 10.1128/spectrum.00513-26

**Published:** 2026-07-29

**Authors:** Jeroen Leus, Annet Heuvelink, Max Schellekens, Arjen Timmerman, Jolanda Niesink-Broekroelofs, Heleen Zweerus, Rowann Behet-Jansen, Sjaak J. de Wit, Jaap A. Wagenaar, Aldert L. Zomer

**Affiliations:** 1 Demetris DierGezondheid, Duiven, the Netherlands; 2 Royal GD, Deventer, the Netherlands; 3 Division of Infectious Diseases and Immunology, Faculty of Veterinary Medicine, Utrecht University 8125, Utrecht, the Netherlands; 4 Department of Population Health Sciences, Faculty of Veterinary Medicine, Utrecht University 8125, Utrecht, the Netherlands; 5 WHO Collaborating Center for Reference and Research on Campylobacter and Antimicrobial Resistance from a One-Health Perspective / WOAH Reference Laboratory for Campylobacteriosis, Utrecht/Lelystad, the Netherlands; University of Georgia College of Veterinary Medicine, Athens, Georgia, USA

**Keywords:** *Escherichia coli*, whole-genome sequencing, lllumina sequencing, Nanopore sequencing, FTIR, IR Biotyper

## Abstract

*Escherichia coli* is a highly diverse bacterial species that includes avian pathogenic *E. coli* (APEC), one of the most prevalent causative agents of disease in poultry worldwide. Rapid and accurate discrimination of *E. coli* strains is essential for outbreak management, antimicrobial resistance surveillance, and vaccine development. In this study, we compared the performance of Fourier Transform Infrared (FTIR) spectroscopy using the IR Biotyper system with Nanopore and Illumina whole-genome sequencing (WGS) for typing 200 *E. coli* isolates, originating from four poultry rearing farms in the Netherlands. From each farm, we sampled 10 one-day-old meat type rearing chicks, and from every chick, we isolated 5 *E. coli* strains. FTIR clustering showed strong concordance with WGS-based classifications, particularly serotyping and core-genome similarity determined by PopPUNK analysis (Adjusted Rand Index 0.75–0.92). While Nanopore and Illumina sequencing provided the highest genetic resolution, FTIR offered a faster (max 6 vs 12–28 days for 200 isolates) and more cost-effective alternative for assessing clonality. Across all methods, multiple strains were detected per farm, whereas most birds carried a single dominant *E. coli* strain. Our findings demonstrate that FTIR provides a reliable and scalable phenotypic method for rapid strain discrimination in *E. coli*, complementing WGS in diagnostic, surveillance, and epidemiological settings where speed and throughput are critical.

**IMPORTANCE:**

*Escherichia coli* is a major pathogen in poultry and a potential zoonotic risk for humans. Rapid and accurate discrimination of avian pathogenic *E. coli* (APEC) strains is critical for outbreak management, antimicrobial resistance surveillance, and the design of effective autogenous vaccines. In this study, we compared Fourier Transform Infrared (FTIR) spectroscopy with Nanopore and Illumina whole-genome sequencing for strain typing of *E. coli* isolates originating from poultry. The results show that FTIR provides comparable clustering accuracy to genomic approaches at a fraction of the time and costs. This work demonstrates that FTIR can serve as a practical, high-throughput alternative for routine monitoring of *E. coli* in veterinary diagnostics and food safety of poultry meat, enabling faster decision-making and more targeted interventions across the poultry production chain.

## INTRODUCTION

*Escherichia coli* is a bacterial species consisting of a large and diverse group of strains that inhabit the large intestine of humans and other warm-blooded animals (1). *E. coli* can be broadly classified into commensal and pathogenic strains (2), with the latter further being subdivided into intestinal pathogenic and extraintestinal pathogenic pathotypes (3, 4). Among the extraintestinal pathogenic pathotypes, avian pathogenic *Escherichia coli* (APEC) are one of the most prevalent bacterial pathogens affecting poultry and other avian species (5–7). *E. coli* infections affect all sectors of poultry farming including broilers, rearing flocks, breeders, and layers. The financial burden associated with APEC infections and treatment is substantial, with global costs up to hundreds of millions of US dollars annually (6, 7). It is estimated that APEC affects approximately 30% of commercial poultry flocks (2). Despite their impact, a critical gap in knowledge persists: the inability to predict the pathogenicity of *E. coli* strains prior to the manifestation of clinical symptoms in birds. Although prediction models based on bacterial genomic DNA sequences exist, their performance only reaches a predictive value of 73% (7).

The determination of strain diversity and strain relatedness in poultry environments is indispensable for epidemiological purposes. Demonstrating whether infections are related, either between poultry farms and flocks or between poultry and humans, allows the identification of the source of an outbreak and can prevent further transmission by targeted interventions. Determining diversity and relatedness can also contribute to study the spread of antimicrobial-resistant *E. coli*. This is of utmost importance as control strategies concerning *E. coli* in poultry production still heavily rely on antimicrobial treatment.

To reduce antimicrobial treatment, given the global increase in antimicrobial resistance (2, 4, 6), there is an urgent need for improving alternative preventive strategies in controlling *E. coli*, such as the use of commercially available vaccines and autogenous vaccines (8). Current methodologies, whereby only one or a limited number of strains are selected, carry the risk of incorporating non-representative *E. coli* strains into autogenous vaccines or the unnecessary use of commercially available vaccines that target serotypes not present in the flock, potentially leading to the induction of antibodies targeting only a subset of the infecting strains thereby reducing vaccine coverage (8). Similarly, the application of bacteriophages also depends on precise knowledge of the genetic background of the infecting *E. coli* strain (9).

It has been suggested that some APEC strains have the same genotype as strains that cause urinary tract infections (UPEC) in children (10). Aside from investigations triggered by food-borne outbreaks, there is no routine practice in place to determine the types of *E. coli* in poultry and predicting their zoonotic potential. Therefore, the risk of *E. coli* infections deriving from poultry products for consumers remains debated (4, 10, 11).

In summary, rapid and accurate insight in the genetic relatedness is important for discrimination of APEC from non-pathogenic *E. coli* strains, for outbreak management, the prediction of zoonotic potential and transmission, antimicrobial resistance surveillance, and vaccine development.

Various methods have been used to detect or assess the pathogenic potential of *E. coli*, including infection experiments, serotyping, cell culture techniques, and molecular techniques such as polymerase chain reaction (PCR) and whole-genome sequencing (WGS) to detect virulence genes. Serotyping is not a high-throughput technique, and it is laborious. Furthermore, there are strains impossible to serotype as they carry a novel O-antigen cluster for which no antiserum has been developed (5). Pulsed-field gel electrophoresis (PFGE) (12), identification of virulence factors, and multilocus sequence typing (MLST) have been used to find phylogenetic relationships but have limitations (5, 13, 14) regarding resolution. MLST is a marker for the core genome, having an incomplete relation with the phenotype.

To comprehensively understand APEC molecular epidemiology, its population dynamics, and intervention strategies in the poultry production chain, WGS offers a high degree of accuracy by identifying all genes and characteristics of an *E. coli* strain (15). However, WGS can be costly and time-consuming and is, therefore, unsuitable for cases requiring rapid results, such as during diagnosis, outbreak investigation, and rapid selection of suitable strains for autovaccine deployment. Finally, costs of sequencing, but also skilled personnel for bioinformatics analysis, are significantly higher than other techniques, making it less deployable for routine diagnostic purposes in flocks exhibiting symptoms of *E. coli* infection.

An alternative method to very rapidly identify strain diversity is Fourier Transform Infrared spectroscopy (FTIR). This method is based on generating an infrared absorption spectrum of bacterial cells, which reflects the unique molecular composition, such as polysaccharides and phospholipids and structural features of the cells (13, 16–19) and has a high correlation with the genome of the bacteria (17–19). For the determination of relatedness, spectral fingerprints of *E. coli* isolates are being analyzed and compared to distinguish between different *E. coli* strains, enabling the identification of clonal relationships.

FTIR analysis has proven to be a successful tool for outbreak investigation during an *E. coli* outbreak in a human hospital (20). To evaluate the applicability of WGS and FTIR in the poultry industry, we compared the ability to discriminate *E. coli* isolates originating from poultry using the IR Biotyper system for FTIR analysis and two WGS techniques, Nanopore and Illumina. We assessed not only their discriminatory ability on different isolates of *E. coli* isolates from Dutch poultry but also hands-on time, time to result, and finally costs.

## MATERIALS AND METHODS

### Sample collection and bacteriological culture

Ten 1-day-old broiler breeder chicks from each of four different farms, less than a day after arrival at the farm, were selected for necropsy. Recently dead or lethargic chicks (suspected to suffer from yolk sac infection or septicaemia by *E. coli*) were chosen. Necropsy was performed at Demetris laboratory (Duiven, the Netherlands). Yolk sacs were sampled using a sterile swab, and swabs were streaked onto MacConkey agar (Bio-Rad, Lunteren, the Netherlands) within a day after necropsy. Following overnight incubation at 37°C, plates were read, and presumptive *E. coli* colonies were biochemically confirmed (indole production in tryptophan broth and Kovac’s reagent) (Tryptophan broth—[Analytichem, Mijdrecht, the Netherlands], Kovac’s reagent [Pro-lab diagnostics, Wirral, UK], 24 h at 41.5°C, adding 3–5 drops of Kovac’s reagent). Following confirmation, *E. coli* isolates (five colonies per plate, i.e., per animal, if available) were stored at −80°C (Pro-lab Diagnostics Microbank yellow box [Pro-lab diagnostics, Wirral, UK]) pending WGS by Nanopore and Illumina sequencing and FTIR analysis by the IR Biotyper system. In Data S1, the full details of each sample are shown.

### Genome sequencing

DNA extraction of *E. coli* was performed using the DNeasy UltraClean Microbial kit from Qiagen. DNA concentrations were checked using a Thermo Fishers Qubit Fluorometer. Illumina sequencing was performed using Illumina Nextseq 500 (Useq, Utrecht sequencing facility) with a maximum read length of 2 × 300 bp. Libraries were prepared with the Illumina Nextera XT DNA Library Preparation Kit according to the manufacturer’s protocol. Read processing and assembly were performed as described for Illumina reads in reference 21. Briefly, reads were processed using trim_galore 0.4.4_dev with options -q 20 --length 250 and assembled using Spades 3.14.1 using default settings. Contigs >200 bp and depth >10 were retained. Nanopore Sequencing was done using the Rapid barcoding Kit (SQK-RBK096) and R9.4.1 flow cells (FLO-MIN106) on the MinION Mk1B (ONT) device. ONT raw reads were subjected to base-calling and demultiplexing using MinKNOW (v4.5.3) with the Super Accurate model. Afterward, the reads were filtered with Filtlong v0.2.1 with option -t 1,200,000,000, and the quality was assessed with FastQC (v0.11.4). Reads were assembled to contigs with Flye v2.9 (22) using default parameters. Assemblies were polished using Medaka 1.4.3 (https://github.com/nanoporetech/medaka) using default settings and Homopolish 0.3.4 (23) using the R9.4 model. Hybrid assemblies were performed using Unicycler v0.5.1 (24) using default settings.

### Genomic analysis

To assess the concordance between WGS platforms and FTIR-based clustering, we constructed a core genome phylogeny including all isolates sequenced with both Illumina and Nanopore technologies. Default parameters were used unless otherwise stated. The quality of all assemblies was checked with Checkm v1.1.3 (25). Serotypes were determined using Ectyper v 1.0.0 (26) and manually corrected using Abricate v 0.8.2 (https://github.com/tseemann/abricate) using the “EcOH” database from 2018 (27) with –min-cov 20. MLST types were determined using mlst v 2.10 (https://github.com/tseemann/mlst). Genome completeness and contamination were determined using CheckM v1.1.3 (25). Core-SNP phylogenetic trees were determined by aligning the genome using Parsnp v.1.2 (28) and visualized using iTOL (29). Strains were determined by using PopPUNK v 1.2.2 (30) *de novo*. SNP distances were determined using pairwise genome alignments of isolates assigned to specific clusters by PopPUNK using nucmer v3.1 using default settings followed by extracting and counting the number of pairwise SNPs using show-snps -CirT, which were visualized using Graphpad Prism 9.

### FTIR

FTIR analysis was performed at the laboratory of Royal GD, Deventer, the Netherlands, using the IR Biotyper system (Bruker Daltonics GmbH & Co. KG, Bremen, Germany). The method used has been described previously (19). In short, *E. coli* isolates were recultured from the stocks stored at −80°C on 6% sheep blood agar (AnalytiChem). Isolates were streaked onto new sheep blood agar plates and incubated at 36°C for 24 h ± 15 min. Then, sample preparation was performed according to the instructions of the manufacturer. Finally, 15 μL of the homogenized suspension prepared per isolate was transferred onto the silicon sample plate device (Bruker PartNo. I23258P) in quadruplicate. Spots were dried at room temperature for 30 min, before loading the plate into the IR Biotyper system. Spectra were acquired and processed by the OPUS software V8.2.28 (Bruker) and the IR Biotyper Client Software V4.0 (Bruker) using manufacturer default settings. IRTS 1 and IRTS 2 (Infrared Test Standard) were measured as quality control prior to sample spectra acquisition, in each run. The analysis of spectra was based on spectra generated within a wavenumber range from 1,300 to 800 cm^−1^. Hierarchical cluster analysis (HCA) applying principal components analysis was done from spectra of the four technical replicates for each isolate, using the Euclidean exploration method with average linkage type using parallelDist v0.2.7, the hclust function in R v4.4.3 (31), and the adj.rand.index function from pdfCluster_v1.0-4. Briefly, clusters were defined by comparing different height cutoffs from the four technical replicates for each isolate using the cut function and comparing the cluster composition against MLST type, O-serotype, and PopPUNK clusters from the hybrid assemblies using the Adjusted Rand Index (ARI) cluster comparison. A value of 0.21 was chosen as being the most discriminative.

## RESULTS

### Sample set description

From four commercial broiler breeder rearing farms (designated Farms A–D), 10 yolk sac samples were collected from recently deceased or lethargic 1-day-old chicks suspected of APEC infection. *E. coli* was isolated from all 40 yolk sac samples, confirming APEC presence across all farms. From each positive sample, five morphologically distinct *E. coli* colonies were selected for further characterization, resulting in a total of 200 isolates. All isolates were subjected to strain typing using FTIR spectroscopy, as well as single runs of WGS with both ONT and Illumina platforms. Isolates failing QC were not repeated (for Nanopore, all the isolates passed QC; for Illumina, five out of the 200 strains had more than 5% contamination).

### Optimization of FTIR clustering thresholds against PopPUNK genomic clones

**Fig 1:**
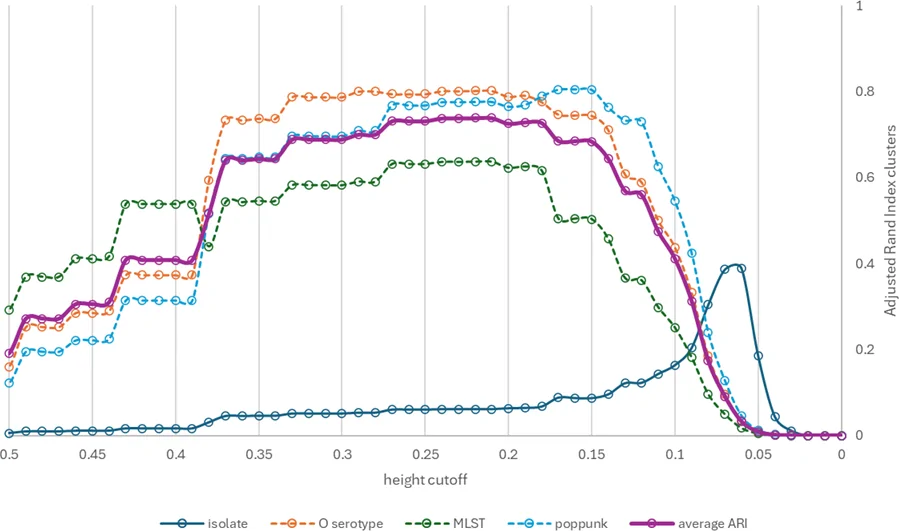
Adjusted Rand Index of FTIR clusters at different height cutoffs compared with sequencing-based clustering.

To determine the optimal height (h) cutoff for defining FTIR-based clusters, we applied hierarchical clustering with average linkage and Euclidean distance to the replicate FTIR spectra of all isolates. Cluster assignments were evaluated across a range of height cutoff values and compared to predicted O serotype, MLST, and PopPUNK genomic clusters from the hybrid assembly and the isolate itself using the Adjusted Rand Index (ARI). As shown in Fig. 1, ARI scores varied across clustering methods, but a consistent optimum was observed in the range of *h* = 0.19 to 0.22, where ARI values were highest and most stable across all data sets. A cutoff of 0.21 was selected as it had the max average ARI. In Data S2, FTIR results and details calculating the cutoff are shown.

### Phylogenetic concordance between sequencing platforms and FTIR clusters

To assess the concordance between WGS platforms and FTIR-based clustering, we constructed a core genome phylogeny including all isolates sequenced with both Illumina and Nanopore technologies showing clustering of isolates across both sequencing platforms, with isolates from the same sequence type grouping tightly regardless of sequencing method (Fig. S1). Each isolate in the phylogenetic tree was additionally color-coded according to its FTIR cluster assignment, MLST type, and PoPPUNK cluster. FTIR clusters aligned well with major phylogenetic clades, supporting the utility of FTIR as a phenotypic proxy for genomic relatedness. Some discrepancies were observed, reflecting the inherently different resolution and underlying molecular basis of FTIR and WGS approaches.

### Multiple strains per farm but predominantly one strain per chicken

To assess the genetic diversity of APEC isolates at both the farm and individual chicken level, we compared strain classifications derived from WGS—including MLST, serotyping, and PopPUNK—as well as from FTIR spectroscopy using the IR Biotyper.

Across the four farms, multiple APEC strains were detected per farm, regardless of the classification method used (Table 1). PopPUNK consistently identified the highest number of distinct strains, suggesting greater discriminatory power compared to MLST and serotyping. For example, Farm A harbored 11–12 strains based on PopPUNK compared to only 5–6 clones by MLST and serotyping. FTIR showed strain counts comparable to those obtained with MLST and serotyping. Comparison between sequencing platforms revealed similar results between Nanopore and Illumina, indicating that both platforms are similar for strain discrimination at the level of resolution offered by MLST, serotyping, and PopPUNK.

**TABLE 1:** Number of strains per farm, identified by different methods of WGS and the IR Biotyper

Farm	WGS									IR Biotyper clusters
	MLST (STs)			Serotypes			PopPUNK clusters			
	Nanopore	Illumina	Hybrid	Nanopore	Illumina	Hybrid	Nanopore	Illumina	Hybrid	
A	6	6	6	5	5	6	11	12	12	6
B	3	3	3	4	4	4	5	5	5	4
C	3	3	3	4	4	4	4	4	4	6
D	7	7	7	9	10	10	10	11	11	8

In this study, we analyzed *E. coli* isolates, originating from infected yolk sacs; at the level of individual chickens, most yolk sacs were colonized by a single APEC strain (Table 2). Using PopPUNK, 32 chickens (out of 40) harbored only 1 strain, while 5 and 2 chickens had 2 or 3 distinct strains, respectively, and 1 animal had 5. MLST and serotyping similarly showed that most chickens carried a single strain although PopPUNK detected more diversity. FTIR results were generally concordant with those from WGS, identifying a single strain in 32 birds, 2 strains in 7 birds, and 3 strains in 1 bird. Although this study is not designed to find how many samples have to be taken in 1-day-old chicks affected by yolk sac infections to predict strain diversity in a bird or in a flock, this result aligns with studies of Kravik et al. (32) and Landman et al. (19).

**TABLE 2:** Number of strains per 1-day-old chicken, identified by different methods of WGS and the IR Biotyper

Strains per chicken	WGS									IR Biotyper
	MLST			Serotype			Poppunk			
	Nanopore	Illumina	Hybrid	Nanopore	Illumina	Hybrid	Nanopore	Illumina	Hybrid	
1	30	31	33	35	33	33	32	32	32	32
2	3	5	5	4	6	6	4	5	5	7
3	1	1	1	1	1	1	4	2	2	1
4	0	0	0	0	0	0	0	0	0	0
5	0	0	0	0	0	0	0	1	1	0

These findings confirm that while multiple strains may co-circulate within farms, individual birds are typically infected with a single dominant strain. PopPUNK was more discriminatory, revealing more diversity, particularly at the farm level.

### Clustering coherence across methods

To evaluate the consistency between clone and cluster assignment derived from different methods, we calculated the ARI for pairwise comparisons (Table 3). Clustering based on serotype showed the highest concordance across all methods. FTIR-based clustering showed high agreement with serotype predictions (ARI = 0.92 and 0.89 for Nanopore and Illumina serotypes, respectively). MLST-derived clusters showed near-perfect agreement between sequencing platforms (ARI = 0.99). In contrast, PopPUNK-based clusters showed slightly lower agreement across methods (e.g., ARI = 0.96 between Nanopore and Illumina). FTIR clustering showed moderate concordance with MLST (ARI range: 0.59–0.63) and high with PopPUNK (ARI range: 0.75–0.77).

**TABLE 3:** Cluster/clone coherence of several WGS methods and IR Biotyper compared with each other: *higher is better*

	ST Nanopore	ST Illumina	ST hybrid	Serotype Nanopore	Serotype Illumina	Serotype hybrid	Nanopore cluster	Illumina cluster	Hybrid cluster	FTIR cluster
ST Nanopore		0.99	1.00	0.62	0.62	0.62	0.58	0.55	0.55	0.63
ST Illumina	0.99		0.99	0.59	0.6	0.6	0.47	0.46	0.46	0.59
ST hybrid	1.00	0.99		0.59	0.59	0.59	0.45	0.43	0.43	0.59
serotype Nanopore	0.62	0.59	0.59		0.97	0.97	0.75	0.72	0.72	0.92
serotype Illumina	0.62	0.6	0.59	0.97		1.00	0.72	0.71	0.71	0.89
serotype hybrid	0.62	0.6	0.59	0.97	1.00		0.73	0.72	0.72	0.89
Nanopore cluster	0.58	0.47	0.45	0.75	0.72	0.73		0.96	0.96	0.77
Illumina cluster	0.55	0.46	0.43	0.72	0.71	0.72	0.96		1.00	0.75
hybrid cluster	0.55	0.46	0.43	0.72	0.71	0.72	0.96	1.00		0.75
FTIR cluster	0.63	0.59	0.59	0.92	0.89	0.89	0.77	0.75	0.75	

### SNP distance variation within strains across platforms

To compare the resolution and internal consistency of strain definitions, we examined the pairwise SNP distances within strains identified by PopPUNK in both Nanopore and Illumina data sets (Fig. S2). For most strains, SNP distances were substantially higher in the Nanopore-based assemblies compared to those from Illumina. This discrepancy is consistent with the higher base-calling error rate with Nanopore sequencing. Despite these differences, strain assignments from both platforms were broadly concordant, indicating that high-level clustering remains robust.

### Comparison of method performance, costs, turnaround, and hands-on time

To evaluate the practical utility of each method, we assessed analytical performance, time to result, hands-on time, and costs for processing 200 APEC isolates using Nanopore sequencing, Illumina sequencing, and the IR Biotyper system (Table 4). Both sequencing platforms yielded high-quality genome assemblies with completeness >95% for nearly all isolates (Nanopore: 198; Illumina: 200) and contamination <5% in all Nanopore and 195 Illumina assemblies. MLST types and serotypes could be determined for the majority of isolates, with Illumina outperforming Nanopore in MLST type assignment (181 vs 145), while serotyping success was slightly higher in Nanopore (184 vs 176). In terms of discriminatory power, PopPUNK identified 26 and 28 genomic clusters from Nanopore and Illumina data, respectively, whereas FTIR resolved 15 clusters. SNP distance analysis revealed a significantly higher mean intra-strain SNP distance in Nanopore data (568 SNPs) compared to Illumina (63 SNPs), with a maximum of 3,915 SNPs observed in Nanopore vs 432 in Illumina when comparing isolates in PopPUNK clusters. Regarding logistics, FTIR offered the fastest time to result (max 6 working days for 200 isolates using 1 device), followed by Nanopore (12 days) and Illumina (28 days). FTIR also had the lowest consumable cost per sample (circa €12), compared to €30 for Nanopore and €73 for Illumina.

**TABLE 4:** Method performance

Criterium	Nanopore	Illumina	Hybrid	IR Biotyper
Completeness >95%	198	200	200	NA^*a*^
Contamination <5%	200	195	195	NA
Serotype predicted	192	189	193	NA
MLST determined	145	181	184	NA
MLST types detected	11	11	11	NA
Serotypes detected	14	14	14	NA
POPPUNK/FTIR Clones detected	26	28	28	15
Mean SNP distance in clone	568	63	63	NA
max SNP distance in clone	3,915	432	432	NA
min SNP distance in clone	15	2	2	NA
Time to result, one device, 200 isolates, maximum unit (working days)	12	28	ND^*b*^	6
Consumable cost per sample (eur) (33)	30	73	ND	12

^*a*^ NA, not available.

^*b*^ ND, not determined.

## DISCUSSION

Accurate strain identification is essential for monitoring and controlling *E. coli* outbreaks (5, 15). Numerous studies have compared methods to achieve rapid, reliable, high-throughput, and cost-effective *E. coli* typing. Across clinical, environmental, and food safety contexts, researchers continue to evaluate and integrate phenotypic, genotypic, and spectroscopic approaches (13, 15, 16, 34, 35).

Our data show that the clusters of *E. coli* detected by FTIR spectroscopy are consistent with MLST and serotype and kmer-based strain clustering using PopPUNK based on Illumina, Nanopore, and hybrid assembled sequencing data. The different methods used each represent different properties of the isolate: across all methods, the highest concordance of FTIR clustering was observed for predicted O-serogroup (Fig. 1, AR 0.86 at h-cutoff of 0.27). FTIR-based clustering at a height cutoff of 0.21, selected for highest average performance for serotype, MLST, and POPPunk clustering, showed high agreement with serotype predictions (ARI = 0.92 and 0.89 for Nanopore and Illumina/hybrid assembly serotypes, respectively). This shows that the FTIR cluster is indicative of one of the major carbohydrate components of the cell, the O-antigen, suggesting that FTIR may serve as a useful proxy for serotyping. This is of particular interest for vaccine selection, where accurate serotype identification is critical.

MLST-derived clusters showed near-perfect agreement between sequencing platforms (ARI = 0.99), confirming platform consistency. In contrast, PopPUNK-based clusters showed slightly lower agreement across methods (e.g., ARI = 0.96 between Nanopore and Illumina), reflecting a higher discriminatory resolution which may be influenced by sequencing errors. FTIR and PopPUNK clustering showed a relatively high concordance (0.75–0.77). PoPPUNK clusters are based on kmers of both the core genome and the accessory genome, thereby providing clustering based on both phylogeny of the strain but also accessory gene content. FTIR clustering showed moderate concordance with MLST (ARI range: 0.59–0.63). MLST is a correlate of the phylogenetic history of the strain, as it is a marker for the core genome. These results indicate that FTIR clustering captures a biologically relevant portion of the genomic diversity of *E. coli* despite its faster and lower-cost nature. These results underscore the potential of FTIR as a complementary tool to genomic approaches, especially for rapid phenotypic clustering in diagnostic or surveillance settings.

We used a higher height cutoff value of 0.21 (Fig. 1) than the cutoff values advised by Bruker (0.15–0.18) after comparing with different strain clustering methods. In another study examining 21 *E. coli* isolates from a single outbreak with the IR Biotyper (19), a height cutoff value of 0.16 was determined by manually inspecting the FTIR clustering graph. The optimal cutoff is depending on application, bacterial species, growth temperature, growth time, and incubation medium. Standardization of these parameters is needed, as Novais et al. (16), Muchaamba et al. (36), and Zendri et al. (37) showed, and this is partly applied in the new Biotyper software update. Adjusting the cutoff value can be necessary if colonies have grown under different circumstances.

For diagnostic use and food safety testing, the IR Biotyper can be accurate enough and fast, and analyses can be done at low costs. Our results are in line with Novais et al. (14) showing FTIR to be a good substitute for WGS in routine laboratories. Several other research groups have concluded that the IR biotyper is a useful tool in outbreak investigations of hospital infections (20, 33, 36, 38–41) and in veterinary settings (42–44).

Using FTIR, the absorption of infrared light by molecular bonds in carbohydrates is measured, which, in the case of *E. coli,* are mostly concentrated in the cell envelope. Several research groups have found that the IR spectrum of the bacterial cell wall is a phenotypic characteristic with a high correlation with the genome (13, 16–19). In combination with a database with pathogenic metadata of the isolates, FTIR and/or WGS can give *E. coli* researchers a diagnostic and epidemiological tool to predict pathogenic potential and epidemiologic relations based on predicted strain relatedness, as shown in several studies (31, 32, 35–40). An example is the study by Marangoni et al. (43) which distinguished multidrug-resistant from susceptible *E. coli* by detecting subtle ESBL-associated biochemical shifts in cell wall proteins and lipids within the 1,800–800 cm⁻¹ FTIR fingerprint region, amplified through feature selection and machine-learning classification. Our study shows the IR Biotyper is useful in the area of predicting potential relatedness of *E. coli* strains originating from 1-day-old chicks with yolk sac infection.

By training machine-learning classifiers on spectra from isolates with known MLST types or serotypes, spectral patterns associated with specific genetic lineages or O-antigens can be identified. Once established, these classifiers can rapidly predict the likely genotype or serotype of new isolates without sequencing, offering a high-throughput and low-cost alternative for strain characterization. This approach can support veterinarians in selecting appropriate autogenous vaccine strains, assist food safety authorities in linking related infections across farms or production chains and researchers in predicting the spread of resistant strains.

In routine monitoring, the substantially lower consumable costs and faster turnaround time of FTIR compared with WGS enable the analysis of larger sample sets in shorter timeframes. Clonality estimates can be obtained within days rather than weeks, allowing timely assessment of strain diversity, for instance for identification of strains for autovaccine development. The analytical software accompanying the IR Biotyper facilitates standardized data processing and clustering, making FTIR accessible to laboratories without advanced bioinformatics capacity. This provides a practical framework for screening *E. coli* isolates for potential transmission between flocks, farm workers, and along the poultry production chain.

FTIR is a method detecting all types of biomolecules, such as polysaccharides and phospholipids. In this way, antigen expression on the bacterial cell walls, such as O and H-antigenes, is displayed by infrared. This has a high correlation with the genome of the bacteria (17–19). For outbreak management and transmission tracking, a higher coherence can be needed, especially when studying specific point mutations, exact genetic characteristics, and strain identity. Both Nanopore and Illumina yielded high-quality genome assemblies with completeness >95% for nearly all isolates in our study. SNP distances were substantially higher in the Nanopore-based assemblies compared to those from Illumina. This discrepancy is consistent with the higher base-calling error rate with Nanopore sequencing. Elevated SNP distances in Nanopore data were strain-specific, suggesting that sequence divergence may be influenced by biological factors such as restriction-modification (RM) systems. Despite the difference in SNP distances, WGS offers the highest resolution and enables studying the exact genome of *E. coli*.

However, if a very rapid result is needed for estimating the number of strains and the relatedness of isolates, FTIR could be a very useful technique. Furthermore, Buberg et al. (4) showed that testing of the genotype alone is not enough to discriminate a pathogenic to a non-pathogenic *E. coli*. Phenotypic testing or metadata analysis is also needed.

As reviewed by Novais et al. (16), Muchaamba et al. (36), and Zendri et al. (37), FTIR has been shown to display greater variability in reproducibility compared with WGS. Consequently, method validation is required for each bacterial species. Consistent results depend on standardized culture conditions—including growth medium, incubation temperature, and duration—as well as uniform instrument settings during spectral acquisition and analysis.

FTIR is a very useful and fast tool to predict (in combination with data analysis and machine learning) the spread of certain characteristics of a strain, such as AMR and pathogenicity, to understand the relatedness of *E. coli* isolates and to support decisions if treatment is necessary. Depending on the exact purpose and accuracy needed, FTIR can be a prescreening method, followed by sequencing by Nanopore and/or Illumina.

### Conclusion

Based on the results of this study, we can conclude that FTIR-based clustering showed a high degree of concordance with predicted serotypes, MLST profiles, and genomic clone assignments derived from k-mer-based PopPUNK analysis. This agreement demonstrates that FTIR captures biologically meaningful variation of *E. coli* isolates reflecting both surface structure composition and underlying genomic relatedness. While WGS provides the highest resolution and enables detection of virulence and resistance genes, FTIR offers a rapid, reproducible, and cost-efficient method to estimate potential clonality and relatedness among isolates.

Depending on the purpose of the analysis, such as outbreak investigation, diversity monitoring within flocks, or preliminary vaccine strain selection, FTIR can, therefore, serve as a practical alternative or complementary approach to sequencing-based methods. Integrating FTIR-derived clustering into genomic and antimicrobial resistance surveillance frameworks could substantially expand routine monitoring capacity, supporting early detection of emerging strains and improving risk assessment across the poultry production chain.

## Data Availability

Illumina short-read sequences and Nanopore long-read sequences are available and have been deposited in NCBI under the BioProject accession number PRJEB104171.

## SUPPLEMENTAL MATERIAL

Data S1 spectrum.00513-26-s0001.xlsx Strain and WGS information.

Data S2 spectrum.00513-26-s0002.xlsx ARI FTIR vs sequence based methods

Fig. S1 spectrum.00513-26-s0003.pdf Core genome phylogeny of Nanopore and Illumina sequencing compared to FT-IR.

Fig. S2 spectrum.00513-26-s0004.docx Pairwise SNP distances within strains identified by PopPUNK in both Nanopore and Illumina data sets.

ASM does not own the copyrights to Supplemental Material that may be linked to, or accessed through, an article. The authors have granted ASM a non-exclusive, world-wide license to publish the Supplemental Material files. Please contact the corresponding author directly for reuse.
