# Supplementary material for: Performance of the IR Biotyper, Nanopore, and Illumina sequencing to discriminate Escherichia coli strains originating from poultry
Source: Microbiol Spectr. 2026 Jul 29;14(9):e00513-26. doi: 10.1128/spectrum.00513-26 (PMC13532302; doi:10.1128/spectrum.00513-26)

Core genome phylogeny of Nanopore and Illumina sequening compared to FT-IR

Legend:
vertically, above the phylogenetic tree, there are, from left to right:

- sample number used in the sequencing process
- technology used (Illumina (orange) or Oxford Nanopore Technologies (ONT) (blue))
- farm number (A to D)
- Cluster found with FTIR
- Strains found with PoPPUNK - ONT
- Strains found with PoPPUNK - Illumina
- MLST result
- O serotype

Please note that the same color in column 4, 5 and 6 means the same result.

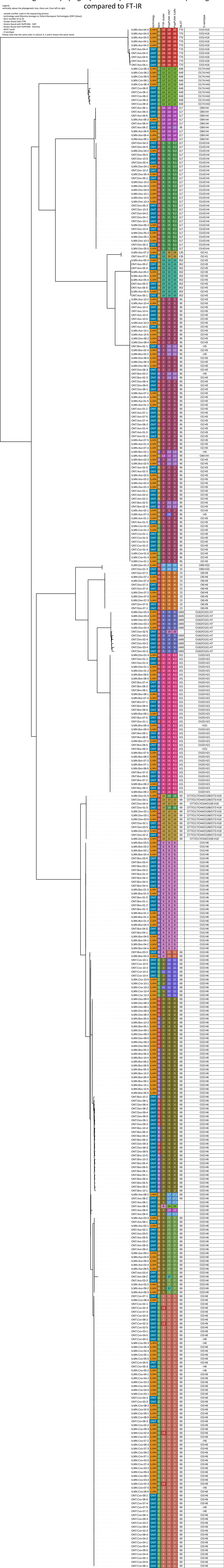

Supplement: Supplementary file 1 [file spectrum.00513-26-s0003.pdf]
