# Supplementary material for: Performance of the IR Biotyper, Nanopore, and Illumina sequencing to discriminate Escherichia coli strains originating from poultry
Source: Microbiol Spectr. 2026 Jul 29;14(9):e00513-26. doi: 10.1128/spectrum.00513-26 (PMC13532302; doi:10.1128/spectrum.00513-26)
Supplement: Supplementary file 2 [file spectrum.00513-26-s0004.docx]

**Supplemental Figure 2. Pairwise SNP distances within strains identified by PopPUNK in both Nanopore and Illumina datasets**

*SNP Distance Variation Within Strains Across Platforms*

To compare the resolution and internal consistency of clone definitions, we examined the pairwise SNP distances within strains identified by PopPUNK in both Nanopore and Illumina datasets (Supplemental Figure 2). For most strains, SNP distances were substantially higher in the Nanopore-based assemblies compared to those from Illumina, with some strains showing intra-clone SNP distances exceeding 4,000 in Nanopore data, versus a maximum of ~400 in Illumina data. This discrepancy is consistent with the higher base-calling error rate with Nanopore sequencing, particularly when using R9.4.1 flow cells. Elevated SNP distances in Nanopore data were clone-specific, affecting only a subset of strains, suggesting that sequence divergence may be influenced by biological factors such as restriction-modification (RM) systems. These systems introduce methylation patterns that can reduce consensus accuracy or alignment quality in long-read data, thereby artificially inflating SNP counts. Despite these differences, clone assignments from both platforms were broadly concordant, indicating that high-level clustering remains robust. However, caution is warranted when using SNP thresholds for fine-resolution typing in Nanopore-only datasets, particularly for strains affected by RM systems or high methylation content.
